# Investigation of 100 SARS-CoV-2 infected families in Wuhan: Transmission patterns and follow-up

**DOI:** 10.7189/jogh.10.021103

**Published:** 2020-12

**Authors:** Urianhkai Horchinbilig, Yanan Gao, Hong Chang, Pengfei Xi, Jinlong Wu, Jun Wang, Wei Liu

**Affiliations:** 1 Department of Gerontology, Inner Mongolia Medical University Affiliated Renmin Hospital, Huhehot Municipality lnner Mongolia Autonomous Region, China; 2 Department of Cardiology, Beijing Anzhen Hospital, Capital Medical University, Beijing, China;; 3 Department of Cardiology, Jilin University Affiliated Chinese Japanese Friendship Hospital, Changchun, Jilin, China; 4 Mongolia Health Committee, Huhehot Municipality lnner Mongolia Autonomous Region, China; 5 Baoshan Hospital, Chifeng City lnner Mongolia Autonomous Region, China

## Abstract

**Background:**

To prevent the spread of severe acute respiratory syndrome coronavirus 2 (SARS-CoV-2), strict control of person-to-person transmission is essential. Family transmission is the most common route of transmission; however, family transmission patterns and outcomes are not well understood.

**Methods:**

We enrolled confirmed cases discharged from Wuhan Zhuankou Fangcang Shelter Hospital from February 17, 2020 to March 8, 2020 along with the family members they had contact with, to evaluate baseline characteristics, family transmission patterns and outcomes. The follow-up period lasted until May 8, 2020.

**Results:**

This study evaluated 369 participants, which included 100 patients admitted to the shelter hospital and the family members they had contact with. Family transmission occurred in 62% of household, with 190 cases confirmed to have SARS-CoV-2 infection. There were eight patterns of family transmission, and spousal transmission (44/83, 53.0%) was the most common pattern, especially in the middle-age generation group (35/83, 42.2%). The homes of the families in which all members were infected had a smaller per capita area than those of other families (29.1 ± 11.89 cm^2^ vs 41.0 ± 19.70 cm^2^, respectively, *P* = 0.037), and the per capita area was negatively associated with the number of infected family members (R = -0.097, *P* = 0.048). Of the 190 confirmed cases, the 113 mild or moderate cases were monitored in fangcang (including Wuhan Zhuankou Fangcang and other fangcang), and the 59 severe cases were treated at designated hospitals. By the end of follow-up, 185 patients recovered and returned home after completing at least 14 days of isolation at the community quarantine center, four died in hospitals, and one died at home before hospitalization. Interestingly, four patients had positive nucleic acid test results after previous negative results, though none of these patients were re-hospitalized, and none of their close contacts reported an infection.

**Conclusions:**

Our data found eight family transmission patterns, of which spousal transmission was the most common. Some patients were also found to have positive test results during follow-up.

On December 29, 2019, several cases of pneumonia caused by a novel coronavirus, recognized as severe acute respiratory syndrome virus 2 (SARS-CoV-2), were reported in Wuhan, Hubei Province, China [1,2]. Since then, the associated disease, coronavirus disease 2019 (COVID-19), was identified and increasingly reported. By April 29, 2020, it had affected more than thirty million patients in 200 countries [3]. The escalating pandemic gained global attention, and the World Health Organization (WHO) increased the risk assessment to a very high level [3]. Many countries locked down and enacted social isolation measures. Previously, China's epidemics have been contained, which may be due to strict control at the community level to prevent person-to-person transmission.

As previous investigations have shown, cluster cases and community transmission of COVID-19 have been common, with family clusters being one of the most common patterns of transmission [4,5]. However, how SARS-CoV-2 is transmitted between family members and the outcomes of infected patients has been largely unknown. In this study, we investigated the characteristics of 100 infected families from Wuhan, the epidemic center of COVID-19 in China, to explore patterns of family transmission and the outcomes of patients with confirmed SARS-CoV-2 infections.

## METHODS

### Participants

Wuhan Zhuankou Fangcang Shelter Hospital was set up as a temporary quarantine location with limited medical resources from February 17, 2020 to March 8, 2020 [6]. During that period, Fangcang provided medical care for nearly 1000 patients. We randomly enrolled (according to the ratio 1:10) 100 confirmed COVID-19 cases and the family members they had contact with. Suspected cases were defined as patients with an epidemiological history and clinical manifestations such as fever and/or respiratory symptoms. Confirmed cases were those suspected cases with positive reverse transcription-polymerase chain reaction (RT-PCR) assays or serology tests for SARS-CoV-2, or they were clinically confirmed cases meeting the diagnostic criteria provided in Diagnosis and Treatment Protocol for Novel Coronavirus Pneumonia (radiological evidence of viral pneumonia with or without a certain epidemiological link; no confirmed virus etiology required) [7,8]. Family members who lived in the same apartment and shared a meal with confirmed cases before hospital admission were defined as having been in contact.

### Clinical characteristics

During the clinical assessment, information including symptoms, coexisting conditions, RT-PCR for SARS-CoV-2 and chest computed tomography (CT) results, and the disease severity of confirmed cases were recorded. Mild or moderate cases were defined as patients presenting with mild symptoms without any severe comorbidities. Severe cases referred to patients with any of the following: breathing rate greater than 30 breaths per min; oxygen saturation level less than 93% at rest; Pao_2_/FiO_2_ (ratio of arterial oxygen partial pressure to fractional inspired oxygen) less than 300 mm Hg; lung infiltrates greater than 50% within 24-48h; severe co-existing conditions; respiratory failure requiring mechanical ventilation; septic shock; or multiple organ dysfunction or failure. Recovery referred to patients meeting all the following criteria: normal temperature for more than three days; marked improvement in respiratory symptoms; decrease in inflammation on pulmonary imaging; and two consecutive negative nucleic acid tests from respiratory tract samples (at least 24-hour interval between samples).

### Transmission characteristics

Family transmission was defined as at least two members having confirmed SARS-Cov-2 infections in one family. Family transmission was determined by examining the relationship between index cases and other infected family members. According to age and marriage status, family members were divided into the young-age generation, middle-age generation and old-age generation group. Considering that some family members presented with an onset of illness at the same time, we could not identify the index cases and therefore used the house effect rate rather than the second infection rate. The household effect rate was calculated as the ratio of infected family members to total family members. The index case was the family member who presented with symptoms at the earliest time and had a final confirmed diagnosis. When two or more cases showed symptoms at the same time, the index case could not be confirmed.

### Data collection

All patients admitted to Wuhan Zhuankou Fangcang Shelter Hospital were asked to complete a questionnaire (including infection condition, symptoms, time of symptom onset, testing results, hospital admissions, and outcomes) to obtain basic information and family transmission data (Supplement). Paper of photo images of all laboratory and imaging tests performed before admission were provided, including those of the family members.

Follow-up continued for two months after discharge, with the last follow-up performed on May 15, 2020. Telephone or WeChat was used for communication between physicians and patients to acquire the latest follow-up information, including repeated RT-PCR and CT results, immunoglobulin (Ig) M and IgG for SARS-CoV-2, rehospitalizations and reinfections. All confirmed cases of COVID-19 discharged from Wuhan Zhuankou Fangcang Shelter Hospital were followed up. Any repeated test results (if available) of the patients and the family members in contact with them were provided to physicians through WeChat in the form of photos.

Data were collected independently by two physicians. All data were extracted manually for quality assurance and validity. When there was a discrepancy between the two physicians, they reviewed the original records to ensure the data were accurate. Statistical analyses were performed by a third person who was not involved in the data-entry process. All patients provided consent to participate in the survey. The Inner Mongolia Health Committee, which dispatched health staff to Wuhan Zhuankou Fangcang Shelter Hospital, approved this study.

### Statistical analysis

Continuous variables were expressed as the mean or median and compared using the Student's t or Mann-Whitney U test as appropriate. Categorical variables were described as frequency rates and percentages and analyzed using the χ^2^ or Fisher exact test as appropriate. Correlation coefficients were calculated for living conditions and family transmission, using the Spearman or Pearson correlation as appropriate. The kappa coefficient was used to test for consistency of the RT-PCR tests and CT scans. A 2-tailed *P* < 0.05 was established as the level of statistical significance for all tests. All analyses were performed using SPSS version 24.0 software (SPSS Inc. Chicago, IL, USA).

## RESULTS

### Demographics and characteristics of infected cases

There was a total of 369 cases included in this study. The family members in contact with 100 patients admitted to the Wuhan Zhuankou Fangcang Shelter Hospital were enrolled, including 190 confirmed to have COVID-19 and 179 who were not infected. The median age was 28 (interquartile range (IQR)  = 13.0-49.8) and 46 (IQR = 38.8-56.0) years in the uninfected group and infected group, respectively (*P* < 0.001). The proportion of children younger than 14 years in infected group was significantly lower than that in the uninfected group (1.0% vs 26.7%, *P* < 0.001). Compared with patients in the uninfected group, those in the infected group also had a higher incidence of hypertension (0.0% vs 0.4%, respectively, *P* = 0.001), diabetes (0.0% vs 3.2%, respectively, *P* = 0.005), cardiovascular disease (1.7% vs 8.5%, respectively, *P* = 0.003) and chronic pulmonary disease (0.0% vs 2.1%, respectively, *P* = 0.021) (**Table 1**).

**Table 1:** Demographics and other characteristics of infected and uninfected subjects*,†

	Uninfected (N = 179)	Infected, (N = 190)	*P*-value
Age (years), median (IQR)	28 (13.0-49.8)	46 (38.8-56.0)	< 0.001‡
-Age <14	46 (26.7%)	3 (1.6%)	< 0.001‡
-Age >70	12 (7.0%)	21 (11.3%)	0.159
Gender:			0.131
-Male	97 (54.2%)	88 (46.3%)	
-Female	82 (45.8%)	102 (53.7%)	
Current smoker	21 (11.7%)	18 (9.5%)	0.831
Previous smoker	1 (0.6%)	4 (2.1%)	0.001‡
Comorbidities:			
-Hypertension	0 (0.0%)	12 (6.4%)	< 0.001‡
-Diabetes	0 (0.0%)	6 (3.2%)	0.005‡
-Cardiovascular disease	3 (1.7%)	16 (8.5%)	0.003‡
-Cerebrovascular disease	2 (1.1%)	0 (0.0%)	0.235
-Chronic pulmonary disease	0 (0.0%)	4 (2.1%)	0.021‡

IQR – interquartile range

*Data are expressed as the n (%) unless otherwise specified.

†Infected and uninfected groups are not adjusted; statistics should be interpreted with caution.

‡Indicates statistical difference.

Of the 190 confirmed cases, 26 (13.7%) were asymptomatic. The most common symptoms found in the symptomatic patients at onset of illness were fever (67.4%), cough (42.6%), and dyspnea (15.8%). Less common symptoms included fatigue and pharyngalgia. In total, 152 patients had positive results on both CT and RT-PCR, showing high consistency between them (R = 0.817, *P* < 0.001). Nineteen patients presented with positive RT-PCR test results and normal CT imaging, whereas 17 patients had abnormal CT results and no RT-PCR confirmation. Moreover, two patients with normal CT and RT-PCR results were confirmed by antibody testing to have SARS-CoV-2 infection. Among the 100 patients discharged from Wuhan Zhuankou Fangcang Shelter Hospital, 98 provided CT reports. Ground glass opacities and fibrous stripes were the most common features of SARS-CoV-2 infection on CT. Nearly 70% of the patients had bilateral infiltration (**Table 2**).

**Table 2:** Baseline characteristics of infected patients*

	Infected patients/total patients (%)
**Symptom:**	
-Asymptomatic	26/190 (13.7%)
-Fever	128/190 (67.4%)
-Cough	81/190 (42.6%)
-Dyspnea	30/190 (15.8%)
-Fatigue	8/190 (4.2%)
-Pharyngalgia	3/190 (1.6%)
**CT manifestation:**	
-Ground glass opacities	85/98 (86.7%)
-Fibrous stripes	8/98 (8.2%)
-Nodule	4/98 (4.1%)
-Pulmonary bulla	4/98 (4.1%)
-Lymph nodes changes	4/98 (4.1%)
-Consolidation	2/98 (2.0%)
-Pleural effusion	2/98 (2.0%)
-Calcification	2/98 (2.0%)
-Interstitial thickening	1/98 (1.0%)
**Affected lobes:**	
-Left upper lobe	57/98 (58.2%)
-Left lower lobe	63/98 (64.3%)
-Right upper lobe	52/98 (53.1%)
-Right middle lobe	44/98 (44.9%)
-Right lower lobe	64/98 (65.3%)
-Bilateral lobes affected	69/98 (70.4%)
**Results of RT-PCR and CT:**	
-Positive RT-PCR + positive CT†	152/190 (80.0%)
-Positive RT-PCR + negative CT	19/190 (10.0%)
-Negative RT-PCR + positive CT†	17/190 (8.9%)
-Negative RT-PCR + negative CT	2/190 (0.1%)

CT – computed tomography, RT-PCR – reverse transcription polymerase chain reaction assay

*Data are expressed as the number ratio (percentage).

†Positive CT refers to ground glass opacity and/or mix consolidation in one or both lobes.

### Family transmission

**Figure 1:**
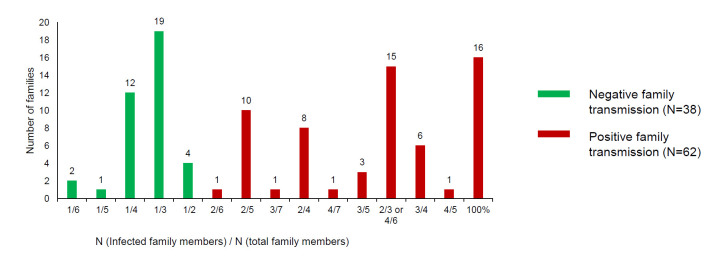
Distribution of house effect rate.

In total, 100 families were analyzed for family transmission patterns. Family transmission occurred in 62 families, of which 16 families were 100% infected and 43 families had more than one infected family members (**Figure 1**).

**Figure 2:**
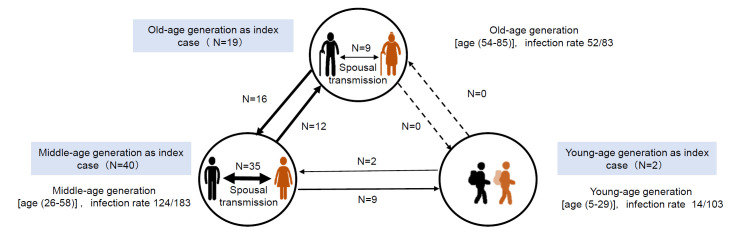
Description of family cluster infections. Line thickness of line reflects the number of infected cases; dotted line indicates no positive family transmission.

As shown in **Figure 2**, 61 patients were identified as index cases, and 83 were diagnosed with SARS-CoV-2 infection contracted from the index cases. In one family, there were 8 infected members and the index case could not be determined. Patients in the middle-age generation group (40/61) were most commonly identified as index cases. More than 60% of the old-age generation (52/83) and middle-age generation groups (124/183) were affected, whereas less than 15% of the young-age generation group (14/103) was infected. There were eight patterns of family transmission. Spousal transmission was the most common pattern (44/83), especially in the middle-age generation group (35/83). In our survey, transmission was not reported between old-age and young-age generations.

We also investigated the relationship between living conditions and family transmission. There were no significant differences in home size (110.8 ± 48.76 cm^2^ vs 102.3 ± 37.26 cm^2^, *P* = 0.367) or per capita area (31.1 ± 16.25 cm^2^ vs 30.5 ± 8.72 cm^2^, *P* = 0.805) between the family transmission and no family transmission groups. However, families with all members infected had a smaller per capita area (29.1 ± 11.89 cm^2^ vs 41.0 ± 19.70 cm^2^, *P* = 0.037) compared to other families, and per capita area was negatively related to the number of infected family members (R = -0.097, *P* = 0.048).

### Clinical courses

**Figure 3:**
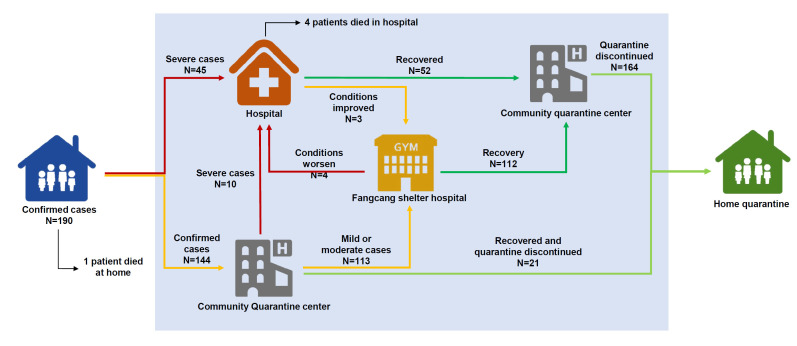
Triage and quarantine management of confirmed cases of COVID-19. Confirmed cases are patients with an epidemiological history and clinical manifestations as well as a positive viral etiology test or pulmonary imaging. Mild or moderate cases are patients who presented with mild symptoms and no severe underlying disease. Severe cases are patients with unstable vital signs or severe chronic comorbidities. Recovery was defined as patients meeting all the following criteria: normal temperature for more than three days; marked improvement in respiratory symptoms; decrease in inflammation on pulmonary imaging; and two consecutive negative nucleic acid tests taken from respiratory tract samples (at least 24-hour interval between samples).

The complete clinical courses of the 190 infected subjects are depicted in **Figure 3**. Forty-five patients were admitted to the designated hospital directly, 144 were initially isolated at the quarantine center, and 113 were transferred to Fangcang (including Wuhan Zhuankou Fangcang Shelter Hospital and other Fangcang) later. Four patients from Fangcang shelter hospitals were referred to designated hospitals due to worsening conditions and three patients were transferred to Fangcang shelter hospitals from designated hospitals when their clinical conditions improved, but the RT-PCR results were still positive. By the end of follow-up, a total of 164 (86.3%) patients were discharged and isolated in the quarantine center, five (2.6%) patients died, including one (male, aged 81 years old) who died at home and four who died during hospitalization (three males and one female, aged 40-79 years old). None of the confirmed cases died at the Fangcang shelter hospital.

### Follow-up

By the end of follow-up, 185 (97.4%) patients had return home from the community quarantine center and RT-PCR and viral serology were reevaluated during home quarantine. Four patients had nasopharyngeal swabs for RT-PCR that were positive after previously negative results, and three presented with persistent positive results of IgM and IgG for SARS-CoV-2 (at 72, 61, and 68 days after the onset of illness). None of these patients were re-hospitalized, and no infections of close contacts were reported. Furthermore, of the 17 patients with CT-confirmed features but without a positive nucleic acid or viral serology test, 8 had negative IgG and IgM results for SARS-CoV-2, indicating that those patients might have had a false positive diagnosis of COVID-19.

## DISCUSSION

To our knowledge, this is the first study to explore transmission patterns of family clusters and outcomes for COVID-19. The study showed that: (1) children have less potential to be infected, whereas individuals with chronic diseases such as hypertension, diabetes, and cardiovascular disease are more likely to be infected by family members; (2) common transmission occurs in more than 60% of families and spousal transmission is the most common; (3) individuals with smaller per capita living areas are more prone to be infected; (4) the Wuhan Zhuankou Fangcang Shelter Hospital supported mild or moderate confirmed cases by providing basic medical resources; and (5) some patients may present with positive RT-PCR results after recovery.

### Home quarantine

Previous studies have reported that family members can be infected with COVID-19 after close contact (eg, attending dinner) with confirmed-positive family members, demonstrating a person-to-person transmission of the virus within the family and providing evidence for asymptomatic carriers as potential sources of SARS-CoV-2 transmission [4,5,9]. SARS-CoV-2 has been shown to have various routes of transmission, the most common of which are respiratory droplets and close contact [10]. Researchers have detected SARS-CoV-2 on the surfaces of objects (eg, table, floor, door handle) in a confirmed case's room and toilet area (eg, sink, toilet bowl) [11]. Moreover, one study revealed that samples from the soles of the shoes of medical were positive for the virus [12]. Compared to other relationships, spouses have closer contact and spend more time together. In our study, spousal transmission was the most common pattern of family transmission.

However, strict home quarantine is still effective in protecting some family members from family transmission, especially during periods of insufficient medical capacity [13]. Suspected or confirmed case should be isolated in a solitary area, and face masks are essential [4]. In addition, some family members may choose to have meals at different times to lower the risk of infection, according to our survey.

Our study also showed that children had a lower risk of infection, which contrasts with the results of a recent study from Shenzhen, China [5]. The reason for this discrepancy might be that: in the epicenter of COVID-19 in Wuhan, some parents managed to send their children to other relatives when they were sick, where the epidemic was not as severe. With respect to the old-age generation, studies have shown that mortality was higher in the elderly population with underlying comorbidities [14]. Therefore, we advise that these patients be admitted to the hospital as early as possible when conditions worsen. Furthermore, this patient population requires the attention of other family members; thus, family transmission is inevitable. In addition, even though asymptomatic carriers tend to be mild, transmission to family members may cause severe COVID-19 pneumonia [15]. Family transmission from asymptomatic carriers occurred in 21 of the 26 carriers in our study, and four of the infected family members had severe disease. Owing to the potential for a worsening condition in these patients or family members, home quarantine alone may be insufficient.

### Fangcang shelter hospital

In our study, one patient died before hospitalization at an early stage of the epidemic due to medical care shortage. After several Fangcang shelter hospitals started admitting patients with mild or moderate symptoms, the number of newly confirmed cases decreased steadily [13,16]. The three characteristics and five essential functions of Fangcang shelter hospitals in Wuhan, which has been well discussed previously [17], made up for the lack of home quarantine. Patients in Fangcang shelter hospitals were well protected by wearing surgical masks, practicing social distancing and performing hand hygiene [17]. In particularly, all patients' stools were specially disposed of by a company to prevent potential transmission [11]. In Wuhan Zhuankou Fangcang Shelter Hospital, where the authors worked, close communication with medical staff provided patients with emotional support. Popular social activities and exercises such as square dancing were also practiced in Fangcang shelter hospitals to maintain basic exercise [17]. Previous studies have demonstrated that regular physical activity is an important component of therapy for most cardiovascular diseases and is associated with reduced cardiovascular and all-cause mortality. Therefore, regular exercise was recommended for all individuals, regardless of whether they had cardiovascular diseases or not [18]. Regular exercise in the Fangcang shelter hospital might have promoted faster rehabilitation. When the Fangcang shelter hospital was suspended, none of the patients had died, and all the medical staff were free from COVID-19, implying that the protective function of the Fangcang shelter hospitals was effective [19].

### Unsettled issues

Our study describes triage and quarantine measures for suspected or confirmed COVID-19 cases, which reflected the full picture of Wuhan (**Figure 3**). However, by the end of follow-up, some issues are still unclear and warrant further study. First, some recovered patients had recurrent positive results of RT-PCR, while there was no evidence suggesting that they were still infectious. It has been demonstrated that virus clearance is associated with the patient's condition, with severe cases having a longer duration of positive RT-PCR results [20]. To date, the longest duration of virus-spillover reported was 37 days [21]; however, some patients in our group had persistent or recurrent positive RT-PCR even longer. Second, six previously confirmed cases with positive CT but negative RT-PCR in our study had negative antibody test results during follow-up, all of whom had been labeled as clinically confirmed cases by the fifth definition. We presume that these COVID-19 diagnose might have been false positives. This is the first time that the number of confirmed cases has been proposed to be potentially overestimated since clinically confirmed COVID-19 cases were counted [8]. However, from the experience of our study, the quarantine measures taken for these patients were still considered to be necessary to prevent possible SARS-CoV-2 transmission even at risk of over-treating false positive cases.

This study has some limitations. First, this is a single-center study with a limited sample size. We only enrolled patients discharged from Wuhan Zhuankou Fangcang Shelter Hospital and tracked their contacted family members, meaning that confirmed cases at a very early stage of the epidemic were not included. Second, information on the distribution of the virus in patients' home was not available, which limited knowledge of the exact routes of family transmission.

## CONCLUSIONS

In conclusion, in this study, children had a lower risk of infection, and individuals with multiple comorbidities were more susceptible. Our study described family transmission patterns, showing that although strict home quarantine prevents SARS-Cov-2 transmission in some families, family transmission was evident in nearly two-thirds of the families, and spousal transmission was the most common pattern. In addition, some patients had recurrent positive RT-PCR results, which requires further investigation.
